# Analysis of carotenogenic genes promoters and WRKY transcription factors in response to salt stress in *Dunaliella bardawil*

**DOI:** 10.1038/srep37025

**Published:** 2017-01-27

**Authors:** Ming-Hua Liang, Jian-Guo Jiang

**Affiliations:** 1 College of Food Science and Engineering, South China University of Technology, Guangzhou, 510640, China

## Abstract

The unicellular alga *Dunaliella bardawil* is a highly salt-tolerant organism, capable of accumulating glycerol, glycine betaine and β-carotene under salt stress, and has been considered as an excellent model organism to investigate the molecular mechanisms of salt stress responses. In this study, several carotenogenic genes (*DbCRTISO, DbZISO, DbLycE* and *DbChyB*), *DbBADH* genes involved in glycine betaine synthesis and genes encoding probable WRKY transcription factors from *D. bardawil* were isolated, and promoters of *DbCRTISO* and *DbChyB* were cloned. The promoters of *DbPSY, DbLycB, DbGGPS, DbCRTISO* and *DbChyB* contained the salt-regulated element (SRE), GT1GMSCAM4, while the *DbGGPS* promoter has another SRE, DRECRTCOREAT. All promoters of the carotenogenic genes had light-regulated elements and W-box *cis*-acting elements. Most WRKY transcription factors can bind to the W-box, and play roles in abiotic stress. qRT-PCR analysis showed that salt stress up-regulated both carotenogenic genes and WRKY transcription factors. In contrast, the transcription levels of *DbBADH* showed minor changes. In *D. bardawil,* it appears that carotenoid over-accumulation allows for the long-term adaptation to salt stress, while the rapid modulation of glycine betaine biosynthesis provides an initial response.

Research on *Dunaliella bardawil* has mainly focused on its salinity tolerance and high β-carotene accumulation under various abiotic stresses (high light, high salinity or nutrient limitation)^1^,^2^. Exploring the stress-induced carotenoid metabolism and elucidating the mechanisms of stress responses and regulation in *D. bardawil* are important for the development of β-carotene production. The carotenoid biosynthesis pathway of *D. bardawil* has been well described by Liang *et al*.^3^. Geranylgeranyl diphosphate (GGPP), the C20 precursor of phytoene, can be synthesized by geranylgeranyl pyrophosphate synthase (GGPS) from three IPP molecules and one DMAPP molecule. The condensation of two GGPP molecules produces phytoene (C40), catalyzed by phytoene synthase (PSY). Then phytoene is desaturated by phytoene desaturases (PDS) and ζ-carotene desaturases (ZDS) and isomerized by 15-cis-ζ-carotene isomerase (ZISO) and carotenoid isomerase (CRTISO) to form the linear *all-trans* lycopene. The cyclation of lycopene by lycopene ε-cyclase (LycE) and lycopene β-cyclase (LycB) introduces ε- and β-ionone end groups, respectively, yielding α-carotene and β-carotene. β-carotene is hydroxylated by β–carotene hydroxylase (ChyB) to produce zeaxanthin.

*D. bardawil* is an impressively salt-tolerant organism, and can adapt to hypersaline environments of up to 5.5 M NaCl. The adaptation of *D. bardawil* to salt stress is attributed to an initial rapid accumulation of glycerol and glycine betaine as intracellular osmoprotectants^4^. Subsequently, the long-term response mechanisms, including salt-induced genes expression, salt tolerance related proteins or transcription factors, allow *D. bardawil* cells to acclimate to high salinity^2^. The regulation of genes related to glycerol metabolism in *Dunaliella salina* under high salt stress conditions has been investigated^5^, and it was suggested that dihydroxyacetone reductase may be a key enzyme in the regulation of glycerol metabolism under salt stress. However, genes involved in glycine betaine metabolism have not yet been studied. In the chloroplast of higher plants, glycine betaine is synthesized from serine via ethanolamine, choline and betaine aldehyde; betaine aldehyde dehydrogenase (BADH) is an important enzyme in the process^6^. Glycine betaine can be accumulated during stress response in many crop plants, including sugarbeet, spinach, barley, wheat and sorghum. However, several plants such as rice, rockcress, tobacco, and potato, naturally cannot synthesize glycine betaine under either stress or non-stress conditions^7^. It has been reported that overexpression of spinach *BADH* in sweet potato (*Ipomoea batatas*) can improve tolerance to various abiotic stresses, including salt, oxidative stress, and low temperature^8^. Thus it is of interest to study *BADH* from *D. bardawil* to reveal the mechanism of salt tolerance and provide tools to potentially engineer salt-tolerant.crop plants.

To investigate the molecular mechanism of salt tolerance in *Dunaliella*, proteomics technology has been used to identify the salt-induced proteins. *Dunaliella* responds to salt stress by improving photosynthetic CO_2_ assimilation and directing carbon and energy resources to glycerol biosynthesis^9^. Another report found that the major salt-regulated proteins were involved in membrane structure stabilization and signal transduction pathways in *D. salina*^10^. Changes in multiple biological process (such as protein synthesis, membrane stabilization, signal transduction, and redox energy production) in response to salt stress suggested that more than one mechanism may play a role in the unique capacity of salt tolerance of *Dunaliella* cells^2^.

It has been reported that *cis*-acting elements and transcription factors also play important roles in plant abiotic stresses^11^,^12^. *cis*-Acting elements exist in the vicinity of the structural portion of a gene, and typically regulate gene transcription by functioning as binding sites for transcription factors. In plants, transcription factors, such as basic leucine-zipper (bZIP), MYB-type, dehydration-responsive-element binding (DREB) proteins and WRKY transcription factors, have been identified and the roles of these transcription factors are still being studied^11^. In algae, MYB-type transcription factors were found to regulate CO_2_-responsive genes and phosphate starvation signaling in *Chlamydomonas reinhardtii*^13^,^14^. In *D. bardawil*, the stress response genes *DbPSY, DbPDS, DbZDS* and *DbLycB* have been isolated, and the *cis*-acting elements in these gene promoters analyzed^15^,^16^.

Recently, the transcriptome sequencing and annotation of *Dunaliella tertiolecta* UTEX LB 999 has been reported^17^, which provides a valuable resource for research on genes encoding key enzymes involved in carotenoid biosynthesis and salt stress regulation. In this study, we investigated the mechanisms of carotenoid accumulation during salt stress in *D. bardawil*. With the help of transcriptome sequencing, key genes (*DbCRTISO, DbZISO, DbLycE* and *DbChyB*) involved in carotenoid biosynthesis, the *BADH* gene involved in glycine betaine biosynthesis, and genes encoding probable WRKY transcription factors were isolated. The transcription levels of these genes were analyzed by quantitative Real-Time PCR (qRT-PCR) during salt stress. The promoters of *DbCRTISO* and *DbChyB* were cloned by genome walking, and the *cis*-acting elements related to salt regulation were analyzed.

## Materials and Methods

### Algal strains and cultivation conditions

The green alga *D. bardawil* strain FACHB-847 was obtained from the Institute of Hydrobiology, Chinese Academy of Science. *D. bardawil* cells were grown in a defined medium^16^ at 26 °C under a 16/8 h light/dark cycle for 7~10 days. In order to study salt stress in *D. bardawil*, algal cells were grown in the defined medium with 0.5 M, 1.0 M, 1.5 M, 2.0 M, 2.5 M, 3.0 M, 3.5 M and 4.0 M NaCl.

### Transcriptome sequencing and CDSs identification

The transcriptome sequencing and annotation of *Dunaliella tertiolecta* UTEX LB 999 (NCBI accession number: SRA023642) has been reported^17^; this can provide a valuable resource for research on genes encoding key enzymes involved in carotenoids biosynthesis and salt stress regulation in this study. We downloaded the two documents SRR070441 and SRR070442 of SRA023642 in NCBI SRA database. The RNA sequencing reads of *D. tertiolecta* UTEX LB 999 were assembled and clustered into 35,625 unigenes. These unigenes were aligned to three public protein databases (Nr, Swiss-Prot and KEGG) by blastx. The Blast2GO software was used to obtain GO annotations for the unigenes. Several coding sequences (CDSs) encoding key enzymes involved in carotenoid biosynthesis (related enzymes like CRTISO, ZISO, LycE and ChyB) and salt stress regulation (related proteins like BADH and WRKY transcription factors) were identified in these unigenes. We used this sequence data to screen *D. bardawil* for related sequences through PCR amplification (testing primers shown in Supplemental Table S1), subcloning and sequencing.

### Genomic DNA isolation and promoters cloning

Genomic DNA extraction from *D. bardawil* in the log or late log phase was performed according to the OMEGA HP Plant DNA Kit. Genome walking was implemented with genome walking kit (Takara). Based on the user manual of the genome walking kit, gene-specific primers (Table 1) were designed to isolate the promoters of *DbCRTISO* and *DbChyB*.

### Bioinformatics Analysis

Sequence similarity analysis was performed using BLAST (http://blast.ncbi.nlm.nih.gov/). The sequence alignment was generated using ClustalX2 software. Conserved domains within a protein or coding nucleotide sequence were detected using the Conserved Domains Search tool (http://www.ncbi.nlm.nih.gov/Structure/cdd/wrpsb.cgi). Translation prediction was performed using the Translation tool (http://web.expasy.org/translate/). The open reading frames (ORFs) in a nucleotide sequence were predicted by ORF Finder (http://www.ncbi.nlm.nih.gov/gorf/gorf.html). Gene structure was displayed by GSDS2.0 (http://gsds.cbi.pku.edu.cn/). Promoter analysis was performed with the PLACE (https://sogo.dna.affrc.go.jp/cgi-bin/sogo.cgi?lang=en&pj=640&action=page&page=newplace) and PlantCARE websites (http://bioinformatics.psb.ugent.be/webtools/plantcare/html/). The database of plant transcription factors is available at the PlantTFDB website (http://planttfdb.cbi.pku.edu.cn/).

### Total RNA extraction and Quantitative Real-Time PCR (qRT-PCR) analysis

The total RNAs were prepared from *D. bardawil* cells cultivated with 0.5 M, 1.0 M, 1.5 M, 2.0 M, 2.5 M, 3.0 M, 3.5 M and 4.0 M NaCl in the late log phase using RNAiso Plus Reagent (Takara). RT-PCR was performed with a 7500 Real-Time PCR System (Applied Biosystems) using the PrimeScript RT Reagent Kit with gDNA Eraser and the SYBR Green PCR Kit (Takara). Primers for quantitative analysis were designed according to the identified CDSs of the corresponding genes and listed in Supplemental Table S2. All PCR reactions were set up in triplicate, and every sample was replicated in parallel three times to ensure statistical relevance. The PCR conditions were used as followed: 30 s at 95 °C, and then 40 cycles of 5 s at 95 °C and 34 s at 60 °C. The specificity of qRT-PCR primers was monitored by the presence of dissociation curves with single peaks and sequencing of the corresponding products with unique bands of the expected sizes. Data were collected and analyzed using SDS software (Applied Biosystems). All results were normalized to the glyceraldehyde-3-phosphate dehydrogenase gene from *D. bardawil* (*DbGAPDH*).

## Results

### Transcriptome analysis and CDSs identification from *D. bardawil*

After PCR amplification, subcloning and sequencing, we found that the coding sequences (CDS) of the genes of interest from *D. bardawil* FACHB-847 were 100% identical to those from *D. tertiolecta* UTEX LB 999. The CDS of all these genes were identified and the details were shown in Table 2. The 1488 bp full-length CDS of *DbCRTISO* encoded 495 Aa, which displayed 60% and 59% sequence similarity with those of *C. reinhardtii* and *Haematococcus pluvialis*, respectively. The partial CDS of *DbZISO* was identified, and the deduced amino acid sequences showed 64% and 62% identities with those of *Ceratophyllum demersum* and *Arabidopsis thaliana*, respectively. Using ORF Finder tool, the Unigene0014471 contained a 1314 bp putative ORF and the Unigene0012559 contained a 954 putative ORF. The 1314 bp putative ORF encoded part of lycopene epsilon cyclase (LycE), with sequence identities of 69% and 64% with those of *H. pluvialis* and *C. reinhardtii*, respectively. The 954 bp putative ORF encoding partial β-carotene hydroxylase (ChyB), displayed 70% and 56% similarities with those of *D. salina* and *C. reinhardtii*, respectively. Previously in our research group, genes such as *GGPS, PSY, PDS, ZDS* and *LycB* have been isolated from *D. bardawil* FACHB-847^3^,^15^,^18^.

A 1496 bp partial *DbBADH* was identified, as the deduced amino acid sequences showed 56% and 55% identities with those of *Triticum aestivum* and *Glycine max*, respectively. According to transcriptome annotations, five unigenes encoding probable WRKY transcription factors were found, but four unigenes were identified (Table 2). Because one unigene putatively encoding the expected DbWRKY5 cannot be cloned according to the testing primers in Supplemental Table S1.

### The features of the probable WRKY transcription factors from *D. bardawil*

WRKY transcription factors play roles in many processes in plants, including regulating many stress reactions^19^. They contain either one or two WRKY protein domains. The WRKY protein domain is a DNA binding domain, about 60 amino acids in length, which contains a highly conserved core WRKYGQ(E)K motif and a zinc-finger region. The cysteine and histidine zinc-finger domain is either CX_4-5_CX_22-23_HXH or CX_7_CX_23_HXC, where X means any amino acid^20^. Most WRKY transcription factors can specifically bind to the W-box *cis*-regulatory element that has a consensus sequence of TTGACC/T^20^.

**Figure 1:**
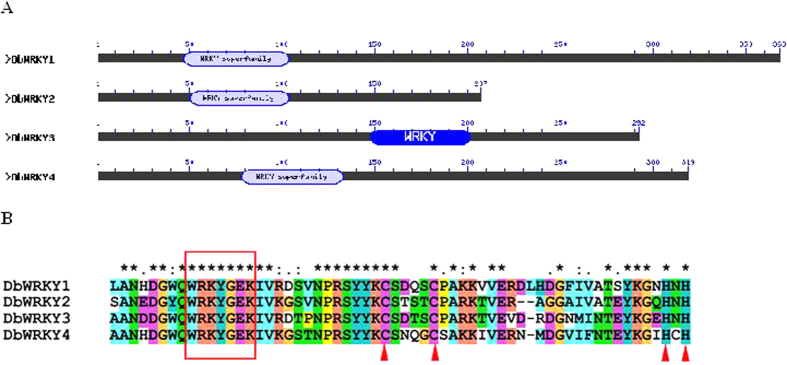
Conserved domain analysis and alignment of four probable WRKY transcription factors from *D. bardawil*. (**A**) Conserved domain analysis of four probable WRKY transcription factors from *D. bardawil*. (**B**) Alignment of the four WRKY protein domains from *D. bardawil* by ClustalX2. The WRKY motif is highlighted in a red frame and the cysteines and histidines that form the zinc-finger structures are shown in red triangles.

Here, four probable WRKY transcription factors were identified from *D. bardawil*, and defined as DbWRKY1, DbWRKY2, DbWRKY3 and DbWRKY4. The conserved domains of the four putative amino acid sequences were predicted by Conserved Domains Search and shown in Fig. 1A. All of them had the WRKYGEK signature motif and a zinc-finger structure at the C-terminus (Fig. 1B). The zinc-finger structure was CX_4_CX_23_HXH in DbWRKY1, CX_4_CX_21_HXH in DbWRKY2, and CX_4_CX_22_HXH in DbWRKY3 and DbWRKY4.

### Promoter cloning and genomic structures of carotenogenic genes

**Figure 2:**
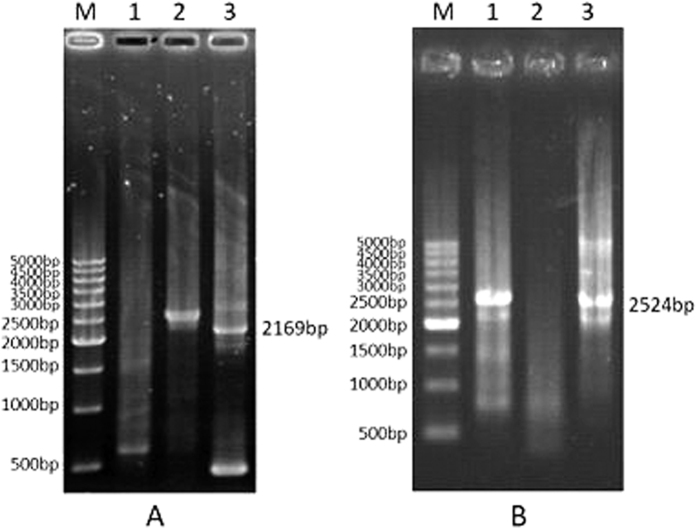
Isolation of promoters of *DbCRTISO* and *DbChyB* in *D. bardawil*. (**A**) Promoter isolation of *DbCRTISO* by genome walking. (**B**) Promoter isolation of *DbChyB* by genome walking. Lane M, 500 bp DNA ladder marker; lanes 1-3, products of 1st PCR, 2nd nested PCR, and 3rd nested PCR, respectively.

We used the full-length CDSs of *DbCRTISO* and *DbChyB* to identify the promoter regions and genomic DNA of *DbCRTISO* and *DbChyB* by genome walking. Sequence analysis by BlastN found that the nucleotide sequence of the third nested PCR product (Fig. 2) possesses identical regions (51 bp in length) as expected with the 5′-*DbCRTISO*, and 37 bp in length with the 5′-*DbChyB*., defining the promoter regions of *DbCRTISO* and *DbChyB* to be 2118 bp and 2487 bp in length, respectively.

**Figure 3:**
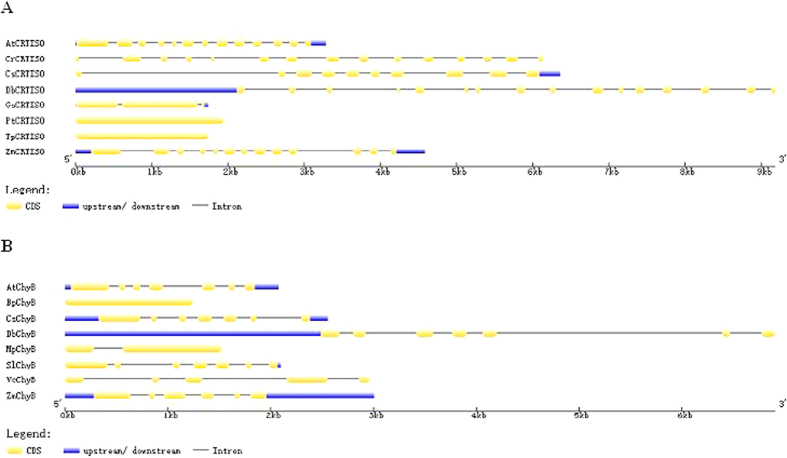
Distribution of exons and introns in the genomic DNA of CRTISOs and ChyBs. (**A**) Distribution of exons and introns in the genomic DNA of *CRTISOs*. The sequences used for analysis are as follows: *CRTISO* from green algae: DbCRTISO (in this study); CrCRTISO, Chlamydomonas reinhardtii (NW_001843787.1); *CsCRTISO, Coccomyxa subellipsoidea* C-169 (NW_005178040.1); *CRTISO* from diatoms: *TpCRTISO, Thalassiosira pseudonana* (NC_012070.1); *PtCRTISO, Phaeodactylum tricornutum* (NC_011675.1); *CRTISO* from red algae: *GsCRTISO, Galdieria sulphuraria* (NW_005178468.1); *CmCRTISO*, Cyanidioschyzon merolae (NC_010140.1); *CRTISO* from higher plants: *AtCRTISO, Arabidopsis thaliana* (NC_003070.9); *ZmCRTISO, Zea mays* (NC_024462.1). (**B**) Distribution of exons and introns in the genomic DNA of *ChyBs*. The sequences used for analysis are as follows: *ChyB* from green algae: *DbChyB* (in this study); *CrChyB, Chlamydomonas reinhardtii* (NW_001843792.1); *BpChyB, Bathycoccus prasinos* (NC_023998.1); *MpChyB, Micromonas pusilla* CCMP1545 (NW_003315882.1); *VcChyB, Volvox carteri* f. nagariensis (NW_003307618.1); *ChyB* from higher plants: *AtChyB, Arabidopsis thaliana* (NW_003302549.1); *CsChyB, Citrus sinensis* cultivar Valencia (NC_023054.1); *SlChyB, Solanum lycopersicum* cultivar Heinz 1706 (NC_015440.2); *ZmChyB, Zea mays* (NC_024468.1).

Analysis of the nucleotide sequences of the cloned genomic DNA fragment indicated that *DbCRTISO* contains 16 exons interrupted by 15 introns, and *DbChyB* has 7 exons and 6 introns (Fig. 3). It appears that the genomic structures of genes from *D. bardawil* are much more complicated (more intron numbers and longer average intron length) than that of the corresponding genes from other algae and plants (Fig. 3). The full-length cDNA and the genomic DNA of *DbGGPS* have been isolated; *DbGGPS* contained 10 exons interrupted by 9 introns and the cloned *DbGGPS* promoter region was 871 bp in length^3^. In our research group, the promoter regions of *PSY, PDS, ZDS* and *LycB* from *D. bardawil* have also been cloned^15^,^16^.

### *cis*-Acting element analysis

Presently, the promoters of seven genes (*DbPSY, DbPDS, DbZDS, DbLycB, DbGGPS, DbCRTISO* and *DbChyB*) involved in carotenoid metabolism in *D. bardawil* have been isolated^3^,^15^,^16^. *cis*-Acting elements of *DbPSY, DbPDS, DbZDS* and *DbLycB* promoters have been analyzed^15^,^16^. Here we comprehensively analyzed several stress-responsive *cis*-acting elements of the seven gene promoters by PLACE and PlantCARE and the results are shown in Table 3. Promoters of *DbPSY, DbLycB, DbGGPS, DbCRTISO* and *DbChyB* genes contained GT1GMSCAM4, salt-regulated elements (SREs), defined as *cis*-acting elements that can control NaCl-induced expression or salt-responsive gene expression. The *DbPDS* promoter did not have any SREs. As well, the *DbGGPS* promoter contained the DRECRTCOREAT motif involved in drought-, high-salt- and cold-responsive gene expression. Exceptionally, only the *DbZDS* promoter contained the hypoosmolarity-responsive element (HRE)^16^. Promoters of the *DbPSY, DbZDS, DbGGPS, DbCRTISO* and *DbChyB* genes contained ASF1MOTIFCAMV, involved in abiotic and biotic stress. All seven of the gene promoters contained light-regulated elements and W-boxes (which might be recognized by WRKY transcription factors).

### qRT-PCR analysis of genes in response to salt stress in *D. bardawil*

**Figure 4:**
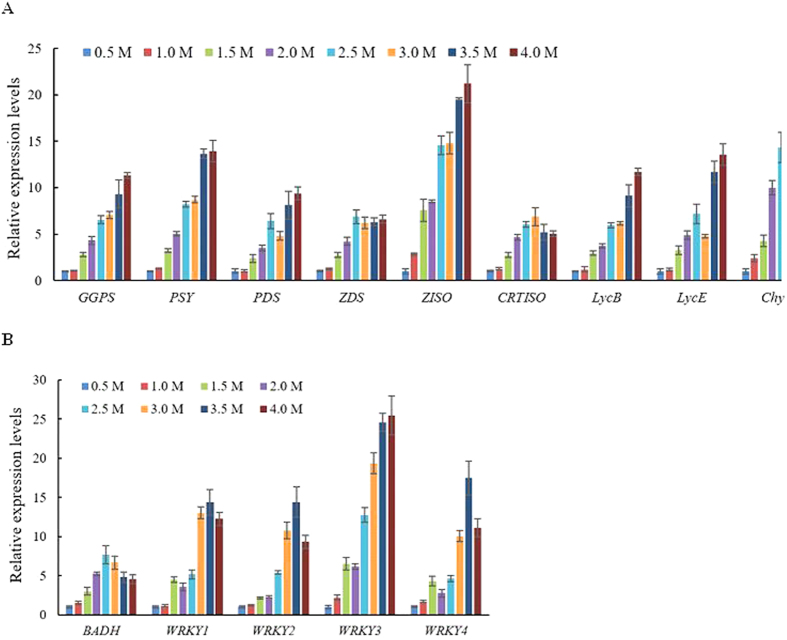
qRT-PCR analysis of genes under salt stress in *D. bardawil.* (**A**) qRT-PCR analysis of the transcription levels of genes involved in carotenoid biosynthesis. (**B**) qRT-PCR analysis of the transcription levels of genes involved in glycine betaine biosynthesis and encoding WRKY transcription factors.

The transcription levels of genes involved in carotenoid biosynthesis, glycine betaine biosynthesis and encoding WRKY transcription factors, were analyzed by qRT-PCR during salt stress. For the carotenoid biosynthesis pathway under salt stress (Fig. 4A), six carotenogenic genes (*DbGGPS, DbPSY, DbZISO, DbLycB, DbLycE* and *DbChyB*) were up-regulated, while the relative expression levels of three carotenogenic genes (*DbPDS, DbZDS* and *DbCRTISO*) were increased slightly. For the glycine betaine biosynthesis pathway under salt stress (Fig. 4B), the relative expression levels of *DbBADH* were increased steadily with the NaCl concentration (from 0.5 M to 2.0 M), and then decreased slightly in the treatments from 2.0 M to 4.0 M NaCl. In addition, it was shown that genes encoding the four probable WRKY transcription factors were up-regulated under salt stress (Fig. 4B).

## Discussion

The transcriptome sequencing and annotation of *D. tertiolecta* UTEX LB 999, provides a valuable resource for research on genes encoding key enzymes involved in carotenoids biosynthesis and salt stress regulation. Recently, the *ZISO* gene was identified in maize^21^. It was confirmed that in higher plants two isomerases (CRTISO and ZISO) are involved in the isomerization that synthesizes *all-trans* lycopene from phytoene^21^,^22^. In this study, these two isomerases were also found in *D. bardawil*, which gave us a new insight into carotenoid metabolism in *D. bardawil*, a halotolerant alga. Currently, a genome sequencing project for *Dunaliella salina* strain CCAP 19/18 is in process^2^. The availability of more genomic data of *Dunaliella* will provide us with a better understanding of the genomic structures of the carotenogenic genes, their gene regulation and their molecular evolution. Presently, we have isolated the genomic DNA of *DbPSY* (6 exons interrupted by 5 introns)^15^, *DbZDS* (12 exons interrupted by 11 introns)^16^, *DbGGPS* (10 exons interrupted by 9 introns)^3^, *DbCRTISO* (16 exons interrupted by 15 introns, Fig. 3A), *DbChyB* (7 exons interrupted by 6 introns, Fig. 3B), and found the genomic structures of these genes were more complex than that from other species. In addition, two subunits of ATP-citrate lyase from *Dunaliella tertiolecta* (DtACLA and DtACLB) were isolated, and there were 12 exons and 11 introns in *DtACLA*, and 20 exons and 19 introns in *DtACLB*^23^. The gene *DsHsp90* from *D. salina* encoding heat shock protein 90 contained 21 exons interrupted by 20 introns^24^. It appears that genes from *Dunaliella* may be intron-rich^24^.

In various biological processes, including abiotic stress responses, hormone responses and developmental processes, the regulation of gene transcription initiation and accurate transcription efficiency can be controlled by *cis*-acting elements in the promoter regions of stress-inducible genes and transcription factors^25^. It appears that several carotenogenic genes (*DbPSY, DbLycB, DbGGPS, DbCRTISO, DbChyB*) in *D. bardawil* were salt stress-inducible as the promoter regions of these genes contained one or more salt-regulated elements (SREs), such as GT1GMSCAM4 and DRECRTCOREAT (Table 3). In addition, salt stress up-regulated most carotenogenic genes in *D. bardawil*, except for *DbZDS* and *DbCRTISO* (Fig. 4A).

In *Haematococcus pluvialis* strains from Queensland, the transcript abundance of seven carotenogenic genes [*IPI*-1, *IPI*-2, *PSY, Lyc*B, *crtR-B* (encoding β-ring hydroxylase), *BKT2* (encoding β-carotene ketolase), and *crtO* (β-carotene oxygenase)] was up-regulated upon salinity stress^26^. And under nutrient stress conditions, carotenogenic genes of *H. pluvialis*, such as *PSY, PDS, LycB, BKT*, and *ChyB* were also up-regulated^27^. In *C. reinhardtii, PSY* and *PDS* displayed a rapid up-regulation in response to light^28^, suggesting that up-regulation of carotenoid biosynthetic pathway genes may occur in response to abiotic stress environments in algae.

All the carotenogenic genes we studied contained the W-box *cis*-acting elements, which can be recognized by WRKY transcription factors. Recently, it has been reported that WRKY transcription factors play key roles in regulating many stress responses in plants^19^. The Plant Transcription Factor Database (PlantTFDB) shows that many more WRKY transcription factors are identified in higher plants than in green algae. There are over 80 WRKY genes in *Arabidopsis thaliana*^29^ and *Oryza sativa*^30^. In green algae, such as *Chlamydomonas reinhardtii, Ostreococcus* sp. RCC809 and *Volvox carteri*, the number of WRKY transcription factors varies from 1 to 3. The evolution of the WRKY transcription factor family between green algae and land plants has been reported^31^. In this study, four probable WRKY transcription factors were identified, and they were up-regulated under salt stress (Fig. 4B). The binding of WRKY transcription factors to W-boxes is a feature of both biotic and abiotic stress responses. It seemed that these four WRKY transcription factors might act as activators under salt stress. These four WRKY transcription factors can be further studied by overexpression in higher plants; it was reported that overexpression of *OsWRKY45* can lead to increased salt and drought tolerance, and enhanced disease resistance^32^, and in *Arabidopsis*, overexpression of either *AtWRKY25* or *AtWRKY33* can result in increased salt tolerance^33^. Intriguingly, compared with the carotenogenic genes, the transcription level changes of *DbBADH* under salt stress were small (Fig. 4B). It appears that carotenoid over-accumulation allows for long-term adaptation to salt stress in *D. bardawil*, and the rapid adjustment of glycine betaine biosynthesis occurs at the start of the salt stress^2^,^4^. The adaptation of *D. bardawil* to salt stress can thus be divided into two stages: first, rapid adjustment of the intracellular concentration of glycerol and glycine betaine, as intracellular osmoprotectants; and second, a long-term response that might include carotenoid accumulation^2^.

Taken together, in *D. bardawil*, promoter regions of stress-inducible genes involved in carotenoid biosynthesis contain *cis*-acting elements, such as SRE, DRE, light-regulated elements, involved in stress-responsive gene expression. Various transcription factors are involved in the regulation of stress-inducible genes. The analysis of *cis*-acting elements and WRKY transcription factors in *D. bardawil* can give us a better understanding of regulatory mechanisms involved in salt stress-responsive gene expression.

## Additional Information

**How to cite this article**: Liang, M.-H. and Jiang, J.-G. Analysis of carotenogenic genes promoters and WRKY transcription factors in response to salt stress in *Dunaliella bardawil. Sci. Rep.* **7**, 37025; doi: 10.1038/srep37025 (2017).

**Publisher's note:** Springer Nature remains neutral with regard to jurisdictional claims in published maps and institutional affiliations.

## Supplementary Material

Supplementary Information

## Figures and Tables

**Table 1:** Primers for the isolation of promoters of *DbCRTISO* and *DbChyB*.

Primers	Specific primers (5′→3′)
P-CRTISO-SP1	ACTTGCAGCCCACGAGCTACCA
P-CRTISO-SP2	CCATCTGTGCAGCACTTGTGAG
P-CRTISO-SP3	CCACTCCACCTCCAATGATGATG
P-ChyB SP1	TCTGATGATGAGGGGCTGGGAAAG
P-ChyB SP2	AGACAGGAGGCAGGAGGGTGAATG
P-ChyB SP3	GTGAACCACCTGTAGCTGTTGTTGC

**Table 2:** The information of the identified genes according to transcriptome sequencing.

Genes	Unigene numbers	Unigene sizes (bp)	Annotations	Verified CDS (bp)	Encoded amino acids (Aa)
*DbCRTISO*	Unigene0009931	2676	Carotenoid isomerase	1488 (Full-length)	495
	Unigene0009932	2711			
	Unigene0009933	2398			
	Unigene0009934	2924			
	Unigene0009935	3202			
	Unigene0009936	3237			
*DbZISO*	Unigene0005835	1533	15-*cis*-ζ-carotene isomerase	1100 (Partial)	365
*DbLycE*	Unigene0014471	2094	Lycopene epsilon cyclase	1314 (Partial)	437
*DbChyB*	Unigene0012559	1179	β-carotene hydroxylase	954 (Partial)	317
*DbBADH*	Unigene0032153	1896	Betaine aldehyde dehydrogenase	1496 (Partial)	497
*DbWRKY1*	Unigene0015734	1107	Probable WRKY transcription factor	1107 (Partial)	368
*DbWRKY2*	Unigene0018100	624	Probable WRKY transcription factor	624 (Partial)	207
*DbWRKY3*	Unigene0018871	1014	Probable WRKY transcription factor	877 (Partial)	292
*DbWRKY4*	Unigene0018871	959	Probable WRKY transcription factor	959 (Partial)	319
*DbWRKY5*	Unigene0015734	1210	Probable WRKY transcription factor	—	—

**Table 3:** Comprehensive analysis of some stress-responsive *cis*-acting elements of seven gene promoters involved in carotenoid metabolism in *D. bardawil*.

Promoters	cis-Acting elements	Functions	References
*DbPSY* promoter	GT1GMSCAM4	NaCl-induced expression	15
	GT1CONSENSUS, BOXIIPCCHS	Light regulation	
	ASF1MOTIFCAMV	Abiotic and biotic stress	
	W-box	Elicitor-responsive element	
*DbPDS* promoter	GATABOX, GT1CONSENSUS, SORLIP1AT	Light regulation	16
	W-box	Elicitor-responsive element	
*DbZDS* promoter	PREATPRODH, GBF5BS	Hypoosmolarity-responsive element (HRE)	16
	GATABOX	Light regulation	
	ASF1MOTIFCAMV	Abiotic and biotic stress	
	W-box	Elicitor-responsive element	
*DbLycB* promoter	GT1GMSCAM4	NaCl-induced expression	16
	GATABOX, CGCGBOXAT, SORLIP1AT	Light regulation	
	W-box	Elicitor-responsive element	
*DbGGPS* promoter	GT1GMSCAM4	NaCl-induced expression	In this study
	DRECRTCOREAT	Drought-, high-salt- and cold-responsive gene expression	
	CBFHV	Dehydration-responsive element (DRE)	
	GATABOX, GT1CONSENSUS, INRNTPSADB, SORLIP2AT	Light regulation	
	ASF1MOTIFCAMV	Abiotic and biotic stress	
	W-box	Elicitor-responsive element	
*DbCRTISO* promoter	GT1GMSCAM4	NaCl-induced expression	In this study
	GATABOX, GT1CONSENSUS, IBOX, INRNTPSADB, SORLIP1AT, TBOXATGAPB	Light regulation	
	ASF1MOTIFCAMV	Abiotic and biotic stress	
	W-box	Elicitor-responsive element	
*DbChyB* promoter	GT1GMSCAM4	NaCl-induced expression	In this study
	CBFHV	DRE	
	GATABOX, GT1CONSENSUS, IBOX, IBOXCORE, INRNTPSADB, SORLIP1AT	Light regulation	
	ASF1MOTIFCAMV	Abiotic and biotic stress	
	W-box	Elicitor-responsive element	
