# Supplementary material for: Analysis of carotenogenic genes promoters and WRKY transcription factors in response to salt stress in Dunaliella bardawil
Source: Sci Rep. 2017 Jan 27;7:37025. doi: 10.1038/srep37025 (PMC5269594; doi:10.1038/srep37025)
Supplement: Supplementary Information [file srep37025-s1.pdf]

Analysis of carotenogenic genes promoters and WRKY transcription factors in response to salt stress in *Dunaliella bardawil*

Ming-Hua Liang, Jian-Guo Jiang*

College of Food Science and Engineering, South China University of Technology, Guangzhou, 510640, China

*Author (Jian-Guo Jiang) for correspondence (*e-mail*: jgjiang@scut.edu.cn; phone: +86-20-87113849; fax: +86-20-87113849).

Supplemental Table S1 Testing primers for isolating the CDS of the corresponding genes.

Genes	Testing Primers	Primers (5'→3')	The length of expected PCR product (bp)
DbCRTISO	T-CRTISO F	ATGGATGCAGAATATGATGCCA	1488
	T-CRTISO R	TCATGGCCACAGCATTGACGCC	
DbZISO	T-ZISO F	CGAGCTTGTGTAGAGCTGCCCT	1100
	T-ZISO R	CTACCATCTCAAATAGAAGCTG	
DbLycE	T-LycE F	ATGTACCTGGCAGCTGAGCTAT	1314
	T-LycE R	TCATGGCTCCGACAGCTCCTTC	
DbChyB	T-ChyB F	ATGGCCTTGTGCGCAACAACAG	954
	T-ChyB R	CTACTGTTGCCTTTCTGCAGGC	
DbBADH	T-BADH F	GTGAATCAGCAAAGAGAGCAGC	1496
	T-BADH R	TCATTGAGGAGCAAGGTCTGGC	
DbWRKY1	T-WRKY1 F	CTGCCCCCAACACCCCCTCCAG	1107
	T-WRKY1 R	GCTGCCGCTCGCGTGGTGCCGG	
DbWRKY2	T-WRKY2 F	GCAGCAGCAGCAGCAGCAGGGG	624
	T-WRKY2 R	CAGCCGGTGCCTATGTGTGAGC	
DbWRKY3	T-WRKY3 F	ATGGCGGAAGCTGTGACTGAAG	877
	T-WRKY3 R	GCTGCTGGTGCTGCTGCTGCTG	
DbWRKY4	T-WRKY4 F	GATGGCCCAGAGGAAGGGCAGC	959
	T-WRKY4 R	TGCTGCTGCTGCTGCTGCTGAA	
DbWRKY5	T-WRKY5 F	CAGCAGCAGCAGCAGCAGCAGG	1210
	T-WRKY5 R	CTGCCACTGCGGCAGCTGCTGC	

Supplemental Table S2 Primers for qRT-PCR used in this study.

Genes	Primers	Forward and reverse primers (5'→3')	Amplicon length (bp)
DbGAPDH	qGAPDH-F	TGCCACCCAGAAGCCTGTAGA	91
	qGAPDH-R	ACCCGTGGAGGAAGGAATG	
DbGGPS	qGGPS-F	TGACTTTGGCTGCCTGTGAA	139

	qGGPS-R	CCTCTGCGGAAGTCATCGTT	
DbPSY	qPSY-F	TACTATCGTGTGGCGGGCA	145
	qPSY-R	CATCTCGCAGAATGTTGGTGA	
DbPDS	qPDS-F	ACATCCCTGCCCCCTGGAATGG	122
	qPDS-R	ATACTTCTGGCCGAAGATGATAGC	
DbZDS	qZDS-F	GCTCCTCCAAAGACCACACCCAG	113
	qZDS-R	GGCTTTGGAGGGGCAGGCTCATC	
DbCRTISO	qCRTISO-F	GCAAATGAGCTTACCAGGCG	95
	qCRTISO-R	CTCCTGTGCGTCTTTGGAGT	
DbZISO	qZISO-F	CCGGGTGATTTTTGCCCTTG	141
	qZISO-R	AAGTTGAGGAGCCACACCAG	
DbLycB	qLycB-F	CAGAAGCCAGAGACCCCTTATTTCAG	133
	qLycB-R	GGCTCTGCAGGTCATGGTACACC	
DbLycE	qLycE-F	TCCAACAGAGGGTTACGCAC	139
	qLycE-R	AGTAGCACACGGCATCATCC	
DbChyB	qChyB-F	AAATGCTCAGACGCAGCAAC	150
	qChyB-R	GCAAGGATGGCTAACCCACT	
DbBADH	qBADH-F	GGAAACCTAGGTGCAGCCAT	136
	qBADH-R	TTCCGCCTAGCTCAAGTGTG	
DbWRKY1	qWRKY1-F	CTGAGCAGTCCTCCACACAG	132
	qWRKY1-R	CCACGTCCATTGCCTCATCT	
DbWRKY2	qWRKY2-F	CACACTCAAGACCGCAAAGC	121
	qWRKY2-R	GTTGCTGCTGGTGTGATCTG	
DbWRKY3	qWRKY3-F	GCGCTTGTCAAACAAGTCCA	140
	qWRKY3-R	CTGCCGTGTTTCCTCCTCTT	
DbWRKY4	qWRKY4-F	GTCAAAGCTGCGACCAAGTG	128
	qWRKY4-R	ATGTTGCCACCCTCACTGTC	
